# The Association between Single-Nucleotide Polymorphisms of *ORAI1* Gene and Breast Cancer in a Taiwanese Population

**DOI:** 10.1100/2012/916587

**Published:** 2012-06-18

**Authors:** Wei-Chiao Chang, Peng Yeong Woon, Yu-Wen Hsu, Shengyu Yang, Yi-Ching Chiu, Ming Feng Hou

**Affiliations:** ^1^Department of Medical Genetics, College of Medicine, Kaohsiung Medical University, Kaohsiung 807, Taiwan; ^2^Cancer Center, Kaohsiung Medical University Hospital, 100 TzYou First Road, Kaohsiung City 807, Taiwan; ^3^School of Pharmacy, College of Pharmacy, Taipei Medical University, Taipei City 110, Taiwan; ^4^Department of Molecular Biology and Human Genetics, Tzu Chi University, Zhongyang Rd., Sec. 3, Hualien 97004, Taiwan; ^5^Department of Tumor Biology, Comprehensive Melanoma Research Center, H. Lee Moffitt Cancer Center, Tampa, FL 12902, USA; ^6^Institute of Clinical Medicine, Kaohsiung Medical University, Kaohsiung, 100 TzYou First Road, Kaohsiung 807, Taiwan; ^7^National Sun Yat-Sen University-Kaohsiung Medical University Joint Research Center, Kaohsiung 807, Taiwan; ^8^Division of General & Gastroenterological Surgery, Department of Surgery, Kaohsiung Medical University Hospital, Kaohsiung 807, Taiwan

## Abstract

*Background*. Breast cancer is the most common malignancy in women. There is increasing evidence suggesting that *ORAI1*, components of store-operated calcium channel, play a pivotal role in breast cancer progression and metastasis. *Methods*. A total of 384 female patients with breast cancer were included in this study. We selected five representative tagging *ORAI1* SNPs from HapMap database with minimum allele frequency (MAF) >10%. Genotyping was performed using TaqMan allelic discrimination assay. Chi-square (*χ* ^2^) test was used to analyze statistical differences among control and patient groups in genotype and allelic frequencies. *Results*. Two of the *ORAI1* SNPs (rs12320939 and rs12313273) were associated with estrogen receptors positive in breast cancer patients under the recessive model. When the Bonferroni correction was performed, the significance still existed. In addition, rs12320939 also associated with the lymph nodal involvement. *Conclusion*. We showed that genetic polymorphisms of *ORAI1* associated strongly with lymph nodal involvement and estrogen receptors (ERs) positive breast cancer patients in a Taiwanese population.

## 1. Background

Breast cancer is the most common female malignancy with an increasing number of new cases around the world [1, 2]. In the United States, the estimated new cases and deaths for female breast cancer patients in 2012 are 226,870 and 39,510, respectively [3]. In recent years, increased incidence rates of breast cancer have been observed in eastern and southeastern Asian women [4]. Epidemiologic studies indicate that patients having a first-degree family history of breast cancer have two-fold increased risk for developing breast cancer compare to the general population [5]. Genetic factor is an important contributor to breast cancer susceptibility. For example, some studies showing genetic variants of *BRCA1* (rs799917) or *Cyclooxygenase-2* (*COX-2*) (rs2745559) have been shown to associate with breast cancer susceptibility [6, 7].

A recent combination of family-based and population-based approaches imply that genes involved in DNA repair (*CHEK2*, *ATM*, *BRIP,* and *PALB2*) are associated with moderate risk in breast cancer subjects [8]. Studies from genomewide association studies (GWASs) in breast cancer reveal SNPs in five genes (*TNRC9*, *FGFR2*, *MAP3K1*, *H19,* and *LSP1*) are associated with breast cancer susceptibility in European population [9]. Hunter et al. (2007) [10] and Stacey et al. (2007) [11] independently replicate the *FGFR2* and *TNRC9* risk alleles in African American population and European descent, respectively. The risk allele locate at the intron 2 of the *FGFR2* gene (rs2981582) represents 5–10% of breast cancer patients with estrogen receptor (ER)-positive tumor, whereas the SNP rs3803662 of *TNRC9* gene seem to be correlated positively with bone metastases and ER-positive breast cancer patients. Similarly, a GWAS breast cancer predisposition with replication and refinement studies involving more than 10,000 case-control identify two more SNPs (rs4415084 and rs10941679) on 5p12 that confer risk, preferentially for ER-positive tumors [12].

Store-operated Ca^2+^ influx is the predominant mechanism of Ca^2+^ entry in nonexcitable cells, such as mast cells, liver cells, and T lymphocytes [13]. Calcium entry through store-operated Ca^2+^ channel has been shown to be important in the regulation of inflammatory reactions in mast cells [14, 15], B cells [16], and cancer cells [17–20]. The cell- and animal-based studies suggested that inhibition of *ORAI1* resulted in stronger focal adhesions and consequently impeded the migration of breast cancer tumor cells [21]. Increasing evidences indicated that about 20% of the cancer candidate genes in breast and colorectal cancers may be adhesion-related genes, suggesting that cell adhesion plays a critical role in cancer progression [22]. Although a recent study has identified the crucial role of *ORAI1* in breast cancer cell migration and metastasis [21], the association between genetic variations of *ORAI1* and the risk of breast cancer is not known. Thus, the aim of this study is to investigate whether genetic variations of *ORAI1* are associated with the histopathological tumor characteristics in Taiwanese breast cancer patients.

## 2. Material and Methods

### 2.1. Sample Collection

384 breast cancer patients were recruited by Cancer Center of Kaohsiung Medical University Hospital. The experimental design was approved by the ethics committee of Kaohsiung Medical University Hospital, and informed consent was obtained from each patient. ER and PR status was analyzed by immunohistochemical staining.

### 2.2. DNA Extraction

Genomic DNA was extracted from whole blood samples in a standard protocol. Whole blood samples from patients were centrifuged at 3000 rpm for 10 min at 4°C. Buffy coat was isolated from the blood samples, and red blood cells (RBCs) were lysed after addition of RBC lysis buffer. DNA was subsequently isolated from blood cells according to manufacturer's procedure.

### 2.3. Genotyping for Five *ORAI1* tSNPs

Genotyping was performed using TaqMan allelic discrimination assay (Applied Biosystems, Foster city, CA, USA). In short, the polymerase chain reactions (PCRs) were performed in a 96-well microtiter plate either with ABI7500 real-time PCR system or ABI9700 Thermal Cycler. The thermal conditions were as follows: denaturing at 95°C for 10 min, followed by 45 cycles of denaturing at 95°C for 15 s, annealing at 60°C for 30 s, and finally extension at 60°C for 1 min. Fluorescence signals from amplicons were further analyzed using the System SDS software version 1.2.3.

### 2.4. Statistical Analysis

Quantitative variables were expressed as mean values with standard deviation (SD). The difference of variable means (e.g., age) between control and patient groups was analyzed by student's *t*-test. Chi-square (*χ* ^2^) test was used to analyze statistical differences among control and patient groups in genotype and allele frequencies. A *P* value of <0.05 was considered statistically significant. Bonfferoni correction was performed to correct multiple testing. Statistical analysis was performed by SAS 9.1 for Windows (SAS Institute Inc., Cary, NC, USA).

## 3. Results

### 3.1. Selection of tSNPs in *ORAI1* for Association Studies

**Figure 1:**
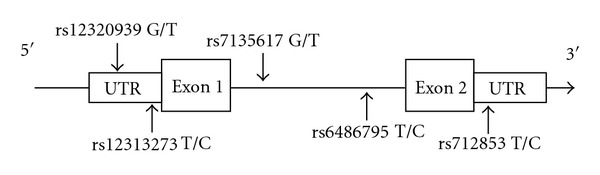
A graphical overview of the genotyped polymorphisms identified in relation to the exon/intron structure of *ORAI1*  gene.

We recruited 384 female breast cancer patients ranging from 28 to 83 years, with a mean age of 51.6 years old. They were predominantly in their middle age, in agreement with the study reported by Shin et al. [4] among Asian female breast cancer patients. Five representative SNPs (rs12313273, rs6486795, rs7135617, rs12320939, and rs712853) with minimum allele frequency (MAF) of >10% were selected from the HapMap Han Chinese database (http://www.hapmap.org/). Two of these SNPs (rs12313273 and rs12320939) were located at *ORAI1* promoter region, rs7135617 and rs6486795 were located at intronic region, and rs712853 was located at the 3′UTR region (Figure 1).

### 3.2. Association between Genetic Polymorphisms of *ORAI1* and Breast Cancer Parameters

Histopathological breast cancer parameters, including tumor recurrence and lymph nodal involvement, were assessed in this study. As shown in Table 1, none of these SNPs were associated with the breast cancer recurrence. When we further tested the association between genotypes and lymph nodal involvement, one of the promoter SNP rs12320939 showed a weak association under recessive model (*P* = 0.03, Table 2). However, the significance disappeared after the Bonferroni correction.

### 3.3. Association of *ORAI1* Polymorphisms with Estrogen Receptor (ER) and Progesterone Receptor (PR)

Hormone receptors ER and PR are overexpressed in the majority of breast cancer patients. In the clinical application, the status of ER and PR are two of the most important prognostic factors [23]. Therefore, we further examined the association between *ORAI1 polymorphisms* and the status of ER or PR hormone receptors in breast cancer patients. As shown in Table 3, a weak association between *ORAI1* (rs12320939; rs7135617) and PR status was found, but the significance disappeared after Bonferroni correction. In addition, rs12320939 and rs12313273 were associated with the ER status under a recessive model (*P* = 0.009; *P* = 0.006) (Table 4). After Bonferroni correction, the significance still can be detected.

## 4. Discussion

Accumulated evidence indicates that Orai1-mediated calcium signaling plays important roles in cancer metastasis. We previously show that calcium influx is critical for cell migration [21, 24]. Abrogation of Orai1-mediated store-operated calcium channel slows down the turnover of focal adhesion and leads to inhibition of metastasis of breast cancer [21]. Orai1 can be activated either by STIM1 in a store-dependent pathway, or by SPCA2 independent of the status of internal calcium store by SPCA2 [25–27]. Interestingly, SPCA2 is overexpressed in the hormone estrogen receptor-positive, luminal subgroup of breast cancer cells [27]. These findings leave open an important issue if the genetic polymorphisms of *ORAI1* convey Ca^2+^ signals to cell proliferative, leading to the pathogenesis of breast cancer. In this study, we provide evidence that *ORAI1* SNP is associated with breast cancer-related parameters in Taiwanese breast cancer patients.

A GWAS study by Stacey et al. [11] involving 1,600 patients and 11,563 controls identified some common variants on chromosomes 2q35 and 16q12 which confer susceptibility to ER-positive breast cancer. Selected SNPs are subsequently genotyped in five replication of European sample sets. They found that two SNPs, rs13387042 on 2q35; rs3803662 on 16q12 consistently associated with breast cancer. Surprisingly, as many as 25% of European population are homozygous for the risk allele A of rs13387042, which put these individuals having an estimated 1.44 fold greater risk than noncarriers. For allele T of rs3803662, about 7% of the European population are homozygous, having a 1.64 fold greater risk of developing breast cancers. However, risk from both alleles is confined only to ER-positive patients. The following year, two more SNPs (rs4415084 and rs10941679) on 5p12 are identified which confer risk in ER-positive tumors. Interestingly, the closest gene, *MRPS30* (also known as PDCD9, programmed cell death protein 9), encodes a component of the small subunit of the mitochondrial ribosome and has previously been implicated playing an important role in apoptosis [12, 28]. Association of breast cancer and these two SNPs has been replicated recently in a 886 cases versus 1089 controls from a African American Women study, in particular the rs4415084 is significantly associated with ER-positive tumors [28]. Most recently, replication of two index SNPs: rs3803662 at 16q12.2/*TOX3* and rs10941679 at 5p12 near *MRPS30* involving 7,800 African American women (including 316 women with incident invasive breast cancer) have also been established [29].

Breast cancer is a complex disease which is influenced by a variety of genetic factors and environmental factors [1, 30]. Numbers of genes including *BRCA1*, *COX-2*, or estrogen receptor [6, 7, 31] have been shown to play critical roles in the development of breast cancer. *COX-2,* which involves in inflammatory, is positively correlated with larger tumor size and higher histological grades in breast cancer samples [32]. Several lines of evidence have indicated that calcium influx through store-operated calcium channel can trigger inflammatory signaling events. In colon cancer or lung cancer, calcium entry through store-operated calcium channel triggers *COX-2* gene activation and expression [18, 20]. Hence, further study is needed to investigate if there is a link between *ORAI1* gene expression and inflammation status of breast cancer patients.

We recognize the potential limitations to our study. It is possible that weak association between *ORAI1* genetic polymorphisms and the risk or tumor-related parameters of breast cancer may be due to the modest sample size, which lead to a small power in statistical analysis. However, sample size may result from international geographic variation of breast cancer incidence [1] and a limited population in Taiwan. It has been demonstrated that Asia has three-fold lower breast cancer incidence rate as compared to North America or Western Europe [1]. Additionally, there were 192,000 estimated cases of breast cancer alone in the United States during 2001 [1] as compared to a total of 22,758 cases in Taiwan collected from 1998 to 2002 [4]. In this regard, our sample size would be reasonably explained in this context. However, we cannot rule out the possibility that there may be other hidden *ORAI1 * SNPs which may associate with breast cancer development. Using direct sequence for *ORAI1* gene in a subset of Han Taiwanese individuals may be helpful to find novel genetic polymorphisms of *ORAI1* that contribute to the development of breast cancer.

## Figures and Tables

**Table 1:** Genotyping and allele frequency of *ORAI1*  gene in patients with breast cancer with or without recurrence.

		Present (%)	Absent (%)		Present (%)	Absent (%)	Dominant	Recessive	Allelic
	Genotype	(*n* = 26)	(*n* = 352)	Allele	(*n* = 26)	(*n* = 352)	*P* value	*P* value	*P* value
rs12320939	TT	2 (8.7)	73 (21.1)	T	20 (43.5)	321 (46.4)	0.4954	0.1524	0.7016
	TG	16 (69.6)	175 (50.6)	G	26 (56.5)	371 (53.6)			
	GG	5 (21.7)	98 (28.3)						
rs12313273	CC	0 (0.0)	22 (6.4)	C	10 (20.0)	182 (26.4)	0.5367	0.1929	0.3206
	CT	10 (40.0)	138 (40.0)	T	40 (80.0)	508 (73.6)			
	TT	15 (60.0)	185 (53.6)						
rs7135617	TT	1 (4.5)	57 (16.9)	T	15 (34.1)	291 (43.2)	0.5685	0.1267	0.2378
	TG	13 (59.1)	177 (52.5)	G	29 (65.9)	383 (56.8)			
	GG	8 (36.4)	103 (30.6)						
rs6486795	CC	1 (4.5)	40 (11.6)	C	15 (34.1)	248 (36.2)	0.7803	0.3055	0.7825
	CT	13 (59.1)	168 (49.0)	T	29 (65.9)	438 (63.8)			
	TT	8 (36.4)	135 (39.4)						
rs712853	CC	5 (20.8)	31 (8.9)	C	21 (43.8)	220 (31.8)	0.2511	0.0577	0.0873
	CT	11 (45.8)	158 (45.7)	T	27 (56.2)	472 (68.2)			
	TT	8 (33.4)	157 (45.4)						

**Table 2:** Genotyping and allele frequency of *ORAI1*  gene in patients with breast cancer with or without nodal involvement.

		Positive (%)	Negative (%)		Positive (%)	Negative (%)	Dominant	Recessive	Allelic
	Genotype	(*n* = 130)	(*n* = 248)	Allele	(*n* = 130)	(*n* = 248)	*P* value	*P* value	*P* value
rs12320939	TT	18 (14.2)	57 (23.6)	T	117 (46.1)	224 (46.3)	0.0688	0.0334*	0.9550
	TG	81 (63.8)	110 (45.4)	G	137 (53.9)	260 (53.7)			
	GG	28 (22.0)	75 (31.0)						
rs12313273	CC	7 (5.5)	15 (6.2)	C	71 (27.7)	121 (25.0)	0.2551	0.7777	0.4196
	CT	57 (44.5)	91 (37.6)	T	185 (72.3)	363 (75.0)			
	TT	64 (50.0)	136 (56.2)						
rs7135617	TT	17 (13.7)	41 (17.4)	T	108 (43.5)	198 (42.1)	0.1997	0.3603	0.7143
	TG	74 (59.7)	116 (49.4)	G	140 (56.5)	272 (57.9)			
	GG	33 (26.6)	78 (33.2)						
rs6486795	CC	12 (9.5)	29 (12.2)	C	94 (37.0)	169 (26.2)	0.2843	0.4304	0.6869
	CT	70 (55.1)	111 (46.6)	T	160 (63.0)	307 (73.8)			
	TT	45 (35.4)	98 (41.2)						
rs712853	CC	11 (8.6)	25 (10.3)	C	81 (31.6)	160 (33.1)	0.8399	0.5918	0.6956
	CT	59 (46.1)	110 (45.5)	T	175 (68.4)	324 (66.9)			
	TT	58 (45.3)	107 (44.2)						

*0.01≦*P*  value < 0.05  is labeled in bold. **P  value < 0.01  is labeled in bold.

**Table 3:** Genotyping and allele frequency of *ORAI1*  gene in patients with breast cancer with different progesterone receptor (PR) status.

		Positive (%)	Negative (%)		Positive (%)	Negative (%)	Dominant	Recessive	Allelic
	Genotype	(*n* = 219)	(*n* = 156)	Allele	(*n* = 219)	(*n* = 156)	*P* value	*P* value	*P* value
rs12320939	TT	52 (24.1)	23 (15.3)	T	210 (48.6)	129 (43.0)	0.6025	0.0416*	0.1343
	TG	106 (49.1)	83 (55.3)	G	222 (51.4)	171 (57.0)			
	GG	58 (26.8)	44 (29.4)						
rs12313273	CC	16 (7.5)	6 (3.9)	C	119 (27.8)	73 (23.9)	0.4110	0.1572	0.2302
	CT	87 (40.6)	61 (39.9)	T	309 (72.2)	233 (76.1)			
	TT	111 (51.9)	86 (56.2)						
rs7135617	TT	31 (14.8)	26 (17.7)	T	166 (39.7)	136 (46.3)	0.0401*	0.4696	0.0819
	TG	104 (49.8)	84 (57.1)	G	252 (60.3)	158 (53.7)			
	GG	74 (35.4)	37 (25.2)						
rs6486795	CC	27 (12.7)	14 (9.4)	C	156 (36.6)	105 (35.2)	0.9220	0.3325	0.7025
	CT	102 (47.9)	77 (51.7)	T	270 (63.4)	193 (64.8)			
	TT	84 (39.4)	58 (38.9)						
rs712853	CC	24 (11.2)	12 (7.8)	C	149 (34.8)	91 (29.7)	0.1976	0.2843	0.1485
	CT	101 (47.2)	67 (43.8)	T	279 (65.2)	215 (70.3)			
	TT	89 (41.6)	74 (48.4)						

*0.01≦*P*  value < 0.05 is labeled in bold.

**Table 4:** Genotyping and allele frequency of *ORAI1*  gene in patients with breast cancer with different estrogen receptor (ER) status.

		Positive (%)	Negative (%)		Positive (%)	Negative (%)	Dominant	Recessive	Allelic
	Genotype	(*n* = 239)	(*n* = 136)	Allele	(*n* = 239)	(*n* = 136)	*P* value	*P* value	*P* value
rs12320939	TT	58 (24.6)	17 (13.1)	T	226 (47.9)	113 (43.5)	0.5870	0.0091**	0.2511
	TG	110 (46.6)	79 (60.8)	G	246 (52.1)	147 (56.5)			
	GG	68 (28.8)	34 (26.1)						
rs12313273	CC	20 (8.5)	2 (1.5)	C	134 (28.6)	58 (21.8)	0.2220	0.0063**	0.0430*
	CT	94 (40.2)	54 (40.6)	T	334 (71.4)	208 (78.2)			
	TT	120 (51.3)	77 (57.9)						
rs7135617	TT	37 (16.2)	20 (15.6)	T	186 (40.8)	116 (45.3)	0.0593	0.8816	0.2413
	TG	112 (49.1)	76 (59.4)	G	270 (59.2)	140 (54.7)			
	GG	79 (34.7)	32 (25.0)						
rs6486795	CC	31 (13.3)	10 (7.8)	C	170 (36.5)	91 (35.3)	0.5586	0.1104	0.7455
	CT	108 (46.4)	71 (55.0)	T	296 (63.5)	167 (64.7)			
	TT	94 (40.3)	48 (37.2)						
rs712853	CC	25 (10.7)	11 (8.2)	C	156 (33.5)	84 (31.3)	0.7459	0.4344	0.5531
	CT	106 (45.5)	62 (46.3)	T	310 (66.5)	184 (68.7)			
	TT	102 (43.8)	61 (45.5)						

*0.01≦*P*  value < 0.05  is labeled in bold. **P  value < 0.01 is labeled in bold.
